# T Cell Receptor Repertoire Analysis Reveals Signatures of T Cell Responses to Human *Mycobacterium tuberculosis*

**DOI:** 10.3389/fmicb.2022.829694

**Published:** 2022-02-07

**Authors:** Ming-Ming Shao, Feng-Shuang Yi, Zhong-Yin Huang, Peng Peng, Feng-Yao Wu, Huan-Zhong Shi, Kan Zhai

**Affiliations:** ^1^Department of Respiratory and Critical Care Medicine, Beijing Institute of Respiratory Medicine and Beijing Chao-Yang Hospital, Capital Medical University, Beijing, China; ^2^Department of Respiratory and Critical Care Medicine, Wuhan Pulmonary Hospital, Wuhan Institute for Tuberculosis Control, Wuhan, China; ^3^Department of Tuberculosis, Nanning Fourth People’s Hospital, Nanning, China

**Keywords:** TCR repertoire, single-cell sequencing, tuberculous patients, polyfunctional CD4^+^ T cells, TRBV4-1

## Abstract

Characterization of T cell receptor (TCR) repertoires is essential for understanding the mechanisms of *Mycobacterium tuberculosis (Mtb)* infection involving T cell adaptive immunity. The characteristics of TCR sequences and distinctive signatures of T cell subsets in tuberculous patients are still unclear. By combining single-cell TCR sequencing (sc-TCR seq) with single-cell RNA sequencing (sc-RNA seq) and flow cytometry to characterize T cells in tuberculous pleural effusions (TPEs), we identified 41,718 CD3^+^ T cells in TPEs and paired blood samples, including 30,515 CD4^+^ T cells and 11,203 CD8^+^ T cells. Compared with controls, no differences in length and profile of length distribution were observed in complementarity determining region 3 (CDR3) in both CD4^+^ and CD8^+^ T cells in TPE. Altered hydrophobicity was demonstrated in CDR3 in CD8^+^ T cells and a significant imbalance in the TCR usage pattern of T cells with preferential expression of TRBV4-1 in TPE. A significant increase in clonality was observed in TCR repertoires in CD4^+^ T cells, but not in CD8^+^ T cells, although both enriched CD4^+^ and CD8^+^ T cells showed T_H_1 and cytotoxic signatures. Furthermore, we identified a new subset of polyfunctional CD4^+^ T cells with CD1-restricted, T_H_1, and cytotoxic characteristics, and this subset might provide protective immunity against *Mtb*.

## Introduction

Since 2007, tuberculosis (TB) has been the leading infectious disease cause of global deaths with 1.4 million deaths reported in 2019 (Furin et al., 2019; World Health Organization [WHO], 2020). Our ability to manage TB as a threat to global health is compromised by deficient elucidation of the immune response that influences the outcomes of *Mycobacterium tuberculosis (Mtb)* infection (Jeyanathan et al., 2018; Tezera et al., 2020). Antigen-specific CD4^+^ T-cell responses play a critical role in the ultimate outcome of *Mtb* infection (Jasenosky et al., 2015; Jeyanathan et al., 2018).

T cells recognize antigen peptide-major histocompatibility complex (MHC) combinations presented on the surface of antigen-presenting cells through T cell receptors (TCRs) (Davis and Bjorkman, 1988; Stubbington et al., 2016). Most TCRs consist of TCRα and TCRβ chains, which are expressed on T cells and recognize antigens by the complementarity determining region 3 (CDR3) (Krogsgaard and Davis, 2005). Recently, structural analysis of the human TCR–CD3 complex assembly has been revealed, providing clues to TCR triggering and laying a foundation for the rational design of diagnostic biomarkers and immunotherapies targeting the complex (Dong et al., 2019). TCR gene transfer was a compelling therapeutic concept that used physiological antigen recognition and T cell activation to generate antigen-specific T cells and provided a way for cancer patients to quickly generate anti-tumor T cells. Meanwhile, the diversity of the TCR library can be used as a predictive biomarker of the immune response of cancer patients. In lymphoma, the V(D)J recombination of TCR could be used as a predictive biomarker of lymphoma risk. These findings were often concentrated in oncology research. In infectious diseases, especially in TB, whether TCR could be used as a diagnostic biomarker or even a target for immunotherapy remains to be further explored (Allam and Kabelitz, 2006; Sadelain et al., 2017; Charles et al., 2020).

Tuberculous pleural effusion (TPE) is the second most common form of extra-pulmonary TB and a common cause of pleural effusions (PEs) in endemic TB areas (Zhai et al., 2016). TPE results from *Mtb* infection of the pleura and is characterized by an intense chronic accumulation of immune cells, especially T cells, in the pleural space (Tong and Shi, 2013). Previous studies have demonstrated that the characteristics of TCR β chain sequence in TPE are different from those in peripheral blood (Li et al., 2014) and that culture filtrate protein 10-specific CD4^+^ and CD8^+^ T cells with biased usage of TCR Vβ9, Vβ12, or Vβ7.2 are presented in TPE (Qiao et al., 2011; Ogongo et al., 2020). In the present study, we performed paired single-cell TCR sequencing (sc-TCR seq) and single-cell RNA sequencing (sc-RNA seq) in TPE and blood samples to depict the characteristics of the length, hydrophobicity, usage pattern and diversity of the CDR3 sequences, and to characterize clonal and transcriptomic profiles in αβT cell subsets in TB patients. Furthermore, we defined a new subset of polyfunctional CD4^+^ T cells with CD1-restricted, T_H_1 and cytotoxic characteristics, which might provide protective immunity against *Mtb*.

## Materials and Methods

### Human Specimens

This study was conducted in accordance with the approved guidelines of the Institutional Review Boards of Beijing Chao-Yang Hospital, Capital Medical University (No. 2018-ke-327), Wuhan Pulmonary Hospital, Wuhan Institute for Tuberculosis Control (No. 2019-1), and Nanning Fourth People’s Hospital [No. (2019)28]. Ten patients with lymphocytic PE, including five TPE patients and five patients with non-TB, malignant PE were enrolled and 20 samples of PE and paired blood were obtained for sc-TCR seq and sc-RNA seq and 40 samples of PE and 12 samples of blood to perform flow cytometry. PE and blood sample preparation and processing methods were followed as previously described (Ye et al., 2012).

### Single-Cell cDNA Library Preparation and Sequencing

According to the manufacturer’s instructions (10x Genomics, Pleasanton, CA, United States), single-cell libraries were constructed using the Single Cell 5′ Library and Gel Bead Kit and the Single Cell V(D)J Enrichment Kit (10x Genomics). The cDNA libraries were constructed using the Single Cell 5′ Library Construction Kit (10x Genomics) and i7 Multiplex Kit (10x Genomics). At least 10 Gb of sequencing data were generated for TCR repertoires, and at least 220 Gb of sequencing data were generated for transcriptome sequencing with NovaSeq 6000 System (Illumina, San Diego, CA, United States) (performed by CapitalBio, Beijing, China).

### Defining T Cells With T Cell Receptor Sequences

Cells with unique productive paired TCRαβ chains remained for subsequent analysis. These were identified based on the following criteria: (1) we excluded cells that only had TCR α or β chain; (2) we retained all cells that had only one paired productive TCR α and β chain; and (3) if a cell had more than one productive αβ chain, the αβ dominant chain was retained.

### Defining T Cell Subsets by Single-Cell mRNA Sequencing

Cell Ranger v.3.0.2 (10x Genomics) was used to perform barcode processing and single-cell gene UMI counting aligned to the GRCh38.93 reference genome. Cells with gene numbers <200 or mitochondrial gene ratios >25% were filtered out. We used canonical marker genes (CD2, CD3D, CD3E, and CD3G) to annotate T cells. Next, T cells were classified as CD4^+^ T cells or CD8^+^ T cells based on gene expression levels of CD4 and CD8A.

### T Cell Receptor Repertoire Analysis

Combining data from sc-TCR seq and sc-RNA seq, we identified the T cells with both unique productive paired TCRαβ sequences and transcriptional profiles for further analysis.

The shape of the TCR distribution was described using parameters defining the Gaussian-like distribution including Kurtosis and Skewness (Peggs et al., 2003). Kurtosis measures the number of events in the central part of the distribution as opposed to the tails and therefore defines the degree of peakedness, and Skewness measures the asymmetry of the distribution above and below the mean (Lee et al., 2016). Hydrophobicity was identified using a modified version of the Kyte-Doolittle index (Kyte and Doolittle, 1982) of average hydrophobicity as previously described (Rechavi et al., 2015).

Expanded clones were defined as those whose αβ chain was shared by at least two cells in a given cell population.

The diversity and clonal expansion using the D50 index corresponds to the percentage of unique sequences that account for 50% of the total number of sequences. Reduced D50 indicates clonal expansions.

We reconstructed TCR sequences and defined two patterns of TCR clonality based on CDR3 sequences in both paired αβ chains: (1) common TCRs were shared across PE and blood samples in the same patient, and (2) enriched TCRs were detected in more than one T cell in a single sample.

### Differential Expression Analysis

Highly enriched T cells were defined as those cells with paired TCRαβ clone number ≥5. Identification of genes differentially expressed between two sets of cells was performed using R package limma, and significance was represented by the Benjamini-Hochberg multiple testing adjustment with *P* ≤ 0.05.

### Flow Cytometry

Cell surface and intracellular staining were performed using antibodies including CD3 (eFluor 450; eBioscience, Waltham, MA, United States; clone UCHT1), CD8a (FITC, eBioscience, clone RPA-T8; or APC-eFluor 780, eBioscience, clone RPA-T8), TRBV4-1 (PE; Miltenyi Biotec, Bergisch Gladbach, Germany; clone REA871), Ghost Dye (Violet 510; Tonbo Biosciences, San Diego, CA, United States), IFN-γ (PE-Cyanine7, eBioscience, clone 4S.B3), T-bet (BV421; BD Biosciences, San Jose, CA, United States; clone O4-46), Granzyme A (PE-Cyanine7, eBioscience, clone CB9), Granzyme B (FITC, BD eBioscience, clone GB11), Granzyme K (eFluor 660, eBioscience, clone G3H69), PRF1 (BV421, BD Biosciences, clone δG9), CCL4 (PerCP-eFluor 710, eBioscience, clone FL34Z3L), CCL5 (eFluor 660, eBioscience, clone VL1), and CXCR3 (PE-Cyanine7, eBioscience, clone CEW33D). Intracellular staining was performed on T cells stimulated with PMA (50 ng/mL; Sigma-Aldrich, St. Louis, MO, United States) and ionomycin (1 mM; Sigma-Aldrich) in the presence of GolgiStop (2/3 μL/mL, BD Biosciences) and Grid-plug (1 μL/mL, BD Biosciences) for 5 h. Flow cytometry was performed on a BD FACSCanto II flow cytometer using BD FACSDiva Software and FCS Express 5 software (*De Novo* Software, Los Angeles, CA, United States).

### Statistical Analysis

A paired *t*-test was used to compare matched samples between pleural fluid and blood assumed to be normally distributed. Student’s *t* test was used to compare the data from sampled pleural fluid or blood between patients with TPE and those with non-TB effusion. Significance was determined if *P* ≤ 0.05.

## Results

### Defining T Cell Subsets With Paired T Cell Receptor Repertoires and Transcriptional Profiling

**FIGURE 1:**
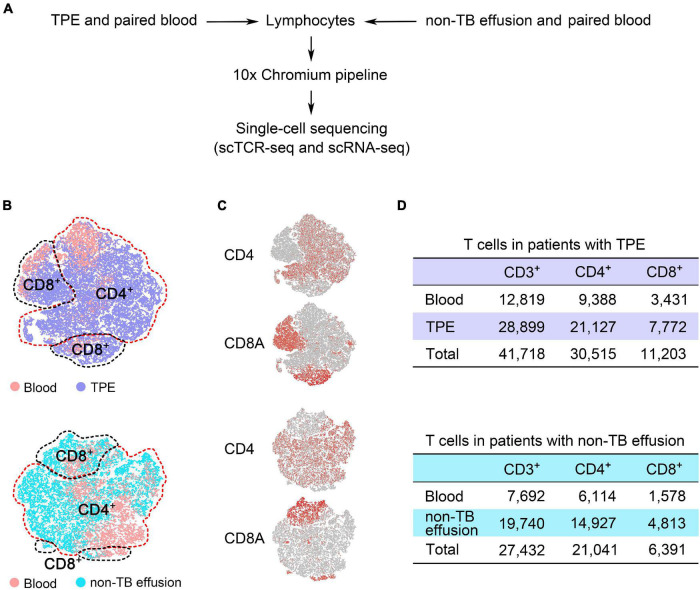
Defining T cell subsets with both unique paired productive TCRαβ sequences and transcriptional profiles from pleural effusion and blood from patients with TPE and those with non-TB effusion. **(A)** Overview of the experimental design. **(B)** t-SNE plots of T cell subsets from TPE patients and non-TB effusion patients (each *n* = 5), with each color-coded for sample type. **(C)** Plots showing the expression of gene markers for CD4^+^ and CD8^+^ T cells defined in panel **(B)**. **(D)** After combining sc-RNA seq and sc-TCR seq data, 41,718 CD3^+^ T cells and 27,432 CD3^+^ T cells were identified in TPE patients and non-TB effusion patients, respectively. TPE, tuberculous pleural effusion; non-TB, non-tuberculosis.

We performed sc-TCR seq on lymphocytes from PE and blood from 10 patients with TPE and those with non-TB effusion and obtained an average of 40,160,537 sequence reads per sample (Supplementary Table 1). After sequencing quality-filtering and combining sc-RNA seq data, we identified 69,150 CD3^+^ T cells, 51,556 CD4^+^ T cells, and 17,594 CD8^+^ T cells with unique paired productive TCRαβ (Figure 1). Among these T cells, 41,718 CD3^+^ T cells, 30,515 CD4^+^ T cells, and 11,203 CD8^+^ T cells were identified in TPE and blood (Supplementary Table 2). None of the mean ratios of CD4/CD3, CD8/CD3, and CD4/CD8 were significantly different between TPE and non-TB effusion (Mann-Whitney *U* test), as well as between pleural fluid and blood samples (Supplementary Table 3).

### Distribution of Complementarity Determining Region 3 Length and Hydrophobicity of T Cell Receptor Repertoires

It is known that CDR3 length is vital in determining the specificity of T cell-mediated immunity because longer CDR3 has a greater potential for sequence variation and can reach into narrow antigenic pockets (Hou et al., 2016). However, no significant differences in amino acid (aa) length and length profile distribution as measured by Kurtosis and Skewness index of CDR3α and CDR3β of CD3^+^, CD4^+^, and CD8^+^ T cells were found in TPE and blood (Supplementary Figure 1). Hydrophobic residues often exist in the protein-protein interfaces, resulting in forming a high affinity of binding, called the hydrophobic effect (Chandler, 2005). Interfacial hydrophobicity of the TCR segment promotes the development of self-reactivity (Stadinski et al., 2016) and antigen recognition (Chowell et al., 2015). We assessed the hydrophobicity of 14 aa (the most frequent length) for CDR3α and CDR3β and found no differences in hydrophobicity were evident in CD4^+^ T cells, but residue 8 was significantly more hydrophobic for CDR3β in the TB site compared with that in the blood (Supplementary Figure 2). Overall, these results suggest that although no differences were found in CDR3α and CDR3β aa length, differences in the biochemical properties of the CDR3β in CD8^+^ T cells in the *Mtb* environment may affect their function.

### Usage Patterns of Variable and Joining Gene Segments of T Cell Receptor Repertoires

The specificity and diversity of TCRs, originating from the rearrangement of variable (V) and joining (J) genes in the α chain and V, diversity (D) and J genes in the β chain, determine the response of CDR3 to MHC complexes and antigenic peptides (Rudolph et al., 2006). Because the TCRβ chain is rearranged before the α chain, we assessed the distribution of TRBV, TRBD, and TRBJ segments of different T cell subsets to define distinctive V, D, and J gene usage of TCRs in TPE patients. Some V gene segments showed significantly higher frequency in TPE than in blood, with TRBV29-1, TRBV18, TRBV27, and TRBV4-2 increased in CD3^+^ T cells, TRBV20-1 increased in CD4^+^ T cells, and TRBV28 increased in CD8^+^ T cells in TPE. Meanwhile, some V gene segments exhibited decreased frequency in TPE, as presented in Table 1. No significant differences in D gene usage of TCRs were observed in TPE compared with blood. Moreover, pleural CD3^+^ T cells showed decreased usage of TRBJ2-3 and increased usage of TRBJ1-3.

**TABLE 1:** Differential expression of TRB V and J genes in T cells between TPE and paired blood*.

Cell type	V gene		J gene	
	Increased	Decreased	Increased	Decreased
CD3^+^ T cells	TRBV29-1, TRBV18, TRBV27, TRBV4-2	TRBV5-1, TRBV25-1, TRBV6-4	NA	TRBJ2-3
CD4^+^ T cells	TRBV20-1	TRBV5-1, TRBV24-1, TRBV5-5, TRBV25-1	NA	NA
CD8^+^ T cells	TRBV28	TRBV12-5, TRBV6-4	TRBJ1-3	NA

*P < 0.05 in each comparison, determined by paired t test. TPE, tuberculous pleural effusion; NA, not available.

**FIGURE 2:**
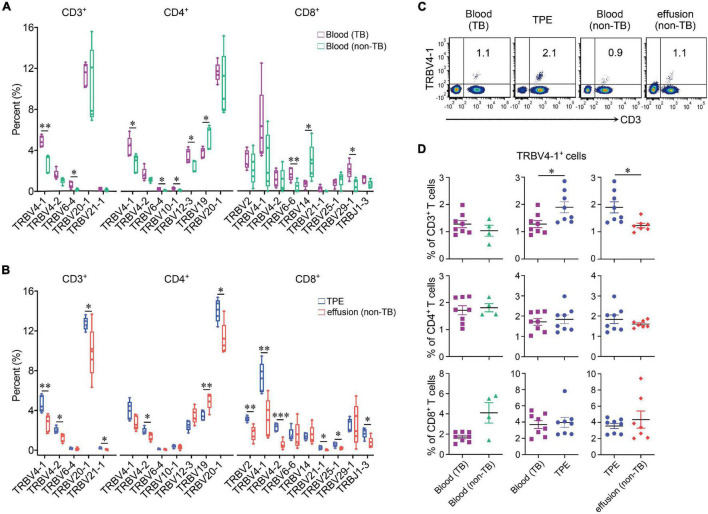
Differential expression of TRB V and J genes in blood and pleural samples from TPE patients and non-TB effusion patients. **(A)** Comparison of usage of TRBV and TRBJ genes in blood between TPE patients and non-TB effusion patients from the single-cell seq data. **(B)** Comparison of usage of TRBV and TRBJ genes in pleural effusion between TPE patients and non-TB effusion patients from the single-cell seq data. **(C)** The representative flow cytometric dot plots of TRBV4-1^+^CD3^+^ T cells in pleural effusion and paired blood from TPE patients and non-TB effusion patients. **(D)** The protein expression of TRBV4-1 in T cell subsets in pleural effusion and the paired blood from TPE patients and non-TB effusion patients by flow cytometry. Data are presented as means ± SEM. *P < 0.05, ^**^P < 0.01, ^***^P < 0.001, determined by Mann-Whitney *U* test. TPE, tuberculous pleural effusion; non-TB, non-tuberculosis.

We also examined the differential expression of TRB V and J genes (Figures 2A,B) by sc-TCR seq in different kinds of PEs and blood, respectively. We noticed that TRBV4-1 significantly increased in TPE patients compared to non-TB effusion patients; for example, TRBV4-1 in CD3^+^ T cells and CD4^+^ T cells in the blood comparison, and TRBV4-1 in CD3^+^ T cells and CD8^+^ T cells in PE comparison. We also noticed a trend of more TRBV4-1 in CD4^+^ T cells in TPE than that in non-TB effusions (*P* = 0.052). To further investigate differential TRBV4-1 changes in T cells after TB infection, we performed flow cytometry to identify TRBV4-1 protein expression. Our findings confirmed that the frequencies of TRBV4-1^+^CD3^+^ cells in TPE were not only much higher than those in blood but also higher than those in non-TB effusions (Figures 2C,D).

### Distribution and Diversity of the Complementarity Determining Region 3 Clonotype of the T Cell Receptor Repertoire

**FIGURE 3:**
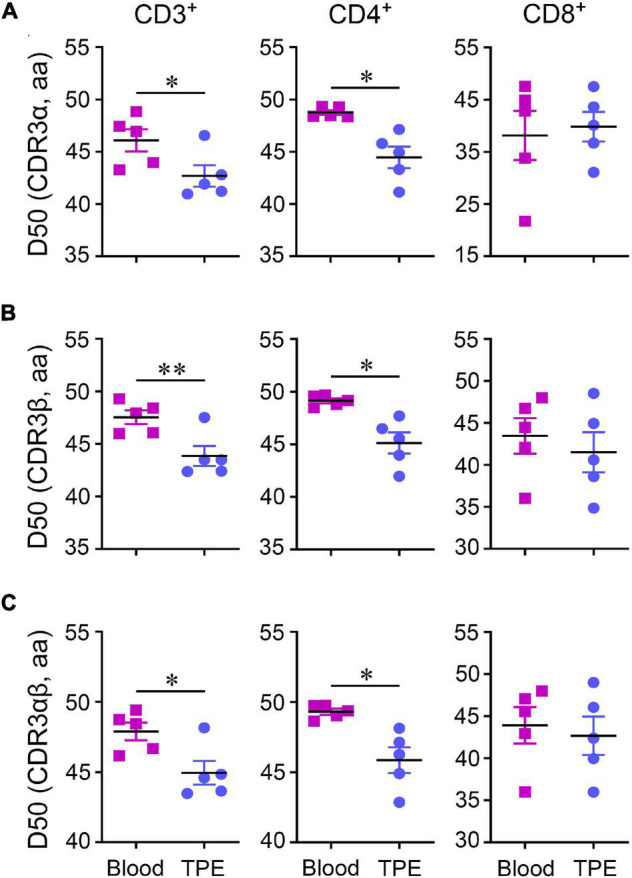
Diversity 50 (D50) index of TCR repertoires in CD3^+^, CD4^+^, and CD8^+^ T cells between human TPE and blood. D50 index in CDR3α **(A)**, CDR3β **(B)**, and CDR3αβ **(C)**. Data are presented as means ± SEM. *P < 0.05, ^**^P < 0.01, determined by paired *t* test. TPE, tuberculous pleural effusion.

Compared to blood, CD4^+^ and CD8^+^ T cell clonal expansion mainly comprised of low-frequency CDR3 clonotypes (<0.05%) and middle-low frequency CDR3 clonotypes (0.05–0.10%) in TPE, respectively. We measured the abnormalities of clonotype frequency in CDR3α, CDR3β and CDR3αβ, and found CDR3α and CDR3β clonotype frequency profiles of CD3^+^, CD4^+^, and CD8^+^ T cells in TPE were similar to the profiles observed in blood (Supplementary Figure 3). However, reduced diversity by the Diversity 50 (D50) index in TCRα, TCRβ and TCRαβ in CD3^+^ T cells and CD4^+^ T cells in TPE indicated clonal expansion existed in TPE (Figure 3).

### T Cell Clonal Expansion in Tuberculosis Patients

**FIGURE 4:**
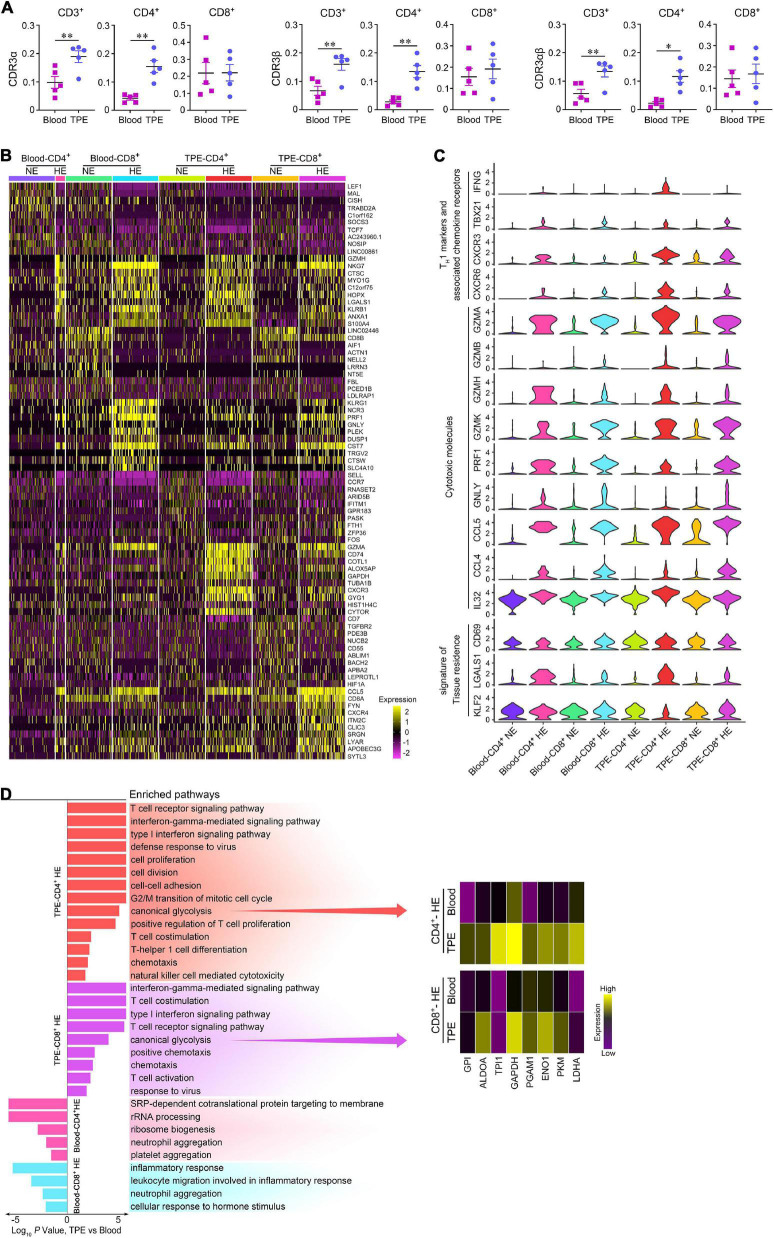
Comparisons of enriched and non-enriched T cell subsets in human TPE and blood. **(A)** Enriched CDR3 aa clonotype frequency comparisons on CD3^+^, CD4^+^, and CD8^+^ T cells between TPE and blood. **(B)** A heatmap comparing the top 10 differentially expressed genes in each highly enriched and non-enriched T cell subsets in 200 randomly sampled cells per cluster, excluding 39 highly enriched CD4^+^ T cells in blood. HE, highly enrichment; NE, non-enrichment. **(C)** Violin plots of expression for select genes in each highly enriched and non-enriched T cell subsets in 200 randomly sampled cells per cluster, excluding 39 highly enriched CD4^+^ T cells in blood. **(D)** Pathway enrichment identified immune response pathways for highly enriched T cell subsets between TPE and blood. Data are presented as means ± SEM. *P < 0.05, **P < 0.01, determined by paired *t* test. TPE, tuberculous pleural effusion; HE, highly enriched; NE: non-enriched.

Next, we performed a detailed analysis of the characteristics of T cell clonal expansion using two patterns of TCR clonality based on CDR3 sequences, named common TCR and enriched TCR. Common TCR analysis showed a trend of more common TCRα and TCRβ in CD4^+^ and CD8^+^ T cells in blood (Supplementary Figure 4). As expected, a significant clonal accumulation of enriched TCRα, TCRβ, and TCRαβ in CD3^+^ T cells was seen in TPE compared to blood (Figure 4A). Interestingly, more enriched TCRs including TCRα, TCRβ, and TCRαβ were observed in TPE-CD4^+^ T cells, but not in TPE-CD8^+^ T cells, suggesting that CD4^+^ cell clonal expansion played an important role in human *Mtb* infection. Considering a significant difference in the fraction of enriched CD4^+^ T cells, but not of common CD4^+^ T cells in TPE vs. blood, we concluded that the increased CD4^+^ T cells present in TPE were might mainly due to local generation and differentiation, and not recruitment from peripheral blood.

We compared the characteristics of gene expression in highly enriched (clone number ≥5) T cell subsets with non-enriched T cell subsets in TPE patients (Figures 4B,C). In TPE and blood, highly enriched CD4^+^ T cells displayed increased expression of the genes related to the T_H_1 such as cells IFNG, TBX21, KLRB1 (Maggi et al., 2012), the T_H_1-associated chemokine receptors CXCR3 (Liu et al., 2005) and CXCR6 (Agostini et al., 2005), and the cytotoxicity markers GZMs, PRF1, NKG7, GNLY, CCL5, CCL4, and IL32 (Montoya et al., 2014; Bai et al., 2015). Similarly, highly enriched CD8^+^ T cells in TPE and blood also exhibited increased expression of the above signatures. Highly enriched CD4^+^ T cells in TPE also expressed signatures of tissue residence, such as increased expression of CD69 (Kumar et al., 2017) and LGALS1 (Szabo et al., 2019), and decreased expression of KLF2 (Masopust and Soerens, 2019).

To further define highly enriched T cell subsets in TB sites, we examined the differentially expressed genes of this subset in TPE using those in blood as controls. We found that highly enriched CD4^+^ and CD8^+^ T cells in TPE upregulated pathways related to TCR signaling, T cell activation, T cell differentiation and proliferation, chemotaxis, response to virus, T_H_1 function, and cytotoxicity (Figure 4D). Of note, we observed that several glycolysis related enzymes were upregulated in highly enriched CD4^+^ T cells and highly enriched CD8^+^ T cells, respectively, and inferred that glycolysis showed significantly enhanced expression in highly enriched T cells in TPE. These results indicated that, compared with blood, highly enriched T cell subsets, especially highly enriched CD4^+^ T cells, consumed more energy to support their T_H_1 function and cytotoxic capacity, and that these cells may adapt to the TB environment to provide protective immunity against *Mtb*.

### Characteristics of TRBV4-1^+^ T Cells in Tuberculous Pleural Effusion

**FIGURE 5:**
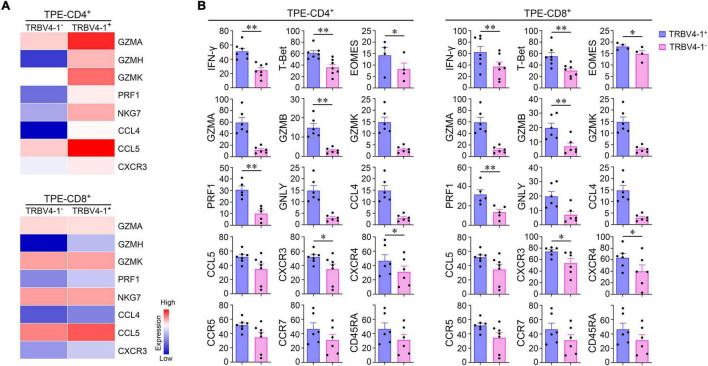
CD1-restricted polyfunctional T cell subsets in human *Mtb* infection site. **(A)** Differential expression of cytotoxic genes from TRBV4-1^+^CD4^+^ T cells, TRBV4-1^+^CD8^+^ T cells, and paired TRBV4-1^–^T cell subsets by scTCR-seq and sc-RNA seq. **(B)** The protein expression of T_H_1 markers and cytotoxic associated molecules on TRBV4-1^+^CD4^+^ T cells, TRBV4-1^+^CD8^+^, and paired TRBV4-1^–^T cell subsets by flow cytometry. *P < 0.05, ^**^P < 0.01, determined by paired *t* test. TPE, tuberculous pleural effusion.

Previously, we found the frequencies of TRBV4-1^+^CD3^+^ cells measured by flow cytometry in TPE were not only much higher than those in blood but also higher than those in non-TB effusions. We analyzed paired sc-TCR seq and sc-RNA seq data, and noticed that both TRBV4-1^+^CD4^+^ and TRBV4-1^+^CD8^+^ T cells highly expressed the genes known to be related to cytotoxicity in the TPE microenvironment (Figure 5A). Li et al. (2014) reported that TRBV20-1 was clonal expansion in TPE. We compared the cytotoxicity-related gene expression of TRBV4-1^+^ T cells and TRBV20-1^+^ T cells in TPE and found that the expressions of these genes in TRBV4-1^+^ T cells were higher than those in TRBV20-1^+^ T cells both in CD4^+^ and CD8^+^ cell population (Supplementary Figure 5; Li et al., 2014). Interestingly, we confirmed that TRBV4-1^+^CD4^+^ and TRBV4-1^+^CD8^+^ T cells highly expressed some of these T_H_1 and cytotoxic transcripts, including IFN-γ, T-bet (encoded by TBX21), CXCR3, GZMA, GZMB, GZMK, PRK1, GNLY, CCL4, and CCL5 in TPE at the protein level (Figure 5B). Given that CD1-restricted T cells preferentially expressed TRBV4-1^+^ TCRs (Reinink et al., 2019), we inferred that polyfunctional CD4^+^ or CD8^+^ T cells with CD1-restricted, T_H_1 and cytotoxic characteristics might provide protective immunity against *Mtb*. Although CD1-restricted IFN-γ-producing CD8^+^ cytotoxic T lymphocytes (CTLs) were reported *in vitro* previously (Busch et al., 2016), we defined a new polyfunctional CD4^+^ T cell subset, CD1-restricted CD4^+^ CTLs with T_H_1 characteristics, in TPE patients in the present study.

## Discussion

Tuberculous pleural effusion is an ideal human disease model for exploring T cell immunity in *Mtb* infection. Recently, Glanville et al. (2017) have developed an algorithm termed GLIPH (grouping of lymphocyte interactions by paratope hotspots) to organize TCRβ sequences into distinct groups of shared specificity either within an individual or across a group of individuals, and to discover T cell antigens through analysis of *Mtb*-specific T cells. GLIPH2 algorithm analyzed 19,044 unique TCRβ sequences with high clustering efficiency and accuracy and identified *Mtb*-specific TCRs including at least five Pro-Pro-Glu proteins serving as targets for T-cell recognition in *Mtb* infection (Huang et al., 2020). However, the landscape of T cells with paired TCRαβ sequences and transcriptional information, which could better discern the “grammar” of TCR recognition, is still unclear. Here, we analyzed CD3^+^, CD4^+^, and CD8^+^ T cells by sc-TCR seq and sc-RNA seq to investigate T cell phenotypes and immune responses in *Mtb* infectious sites.

Our results described the sequence characteristics and clonality of the CDR3 of αβT cells in TPE and identified distinctive signatures of T cell subsets during their immune responses. We found that the TCR regions of CD8^+^ T cells in TPE are thought to form high-affinity binding sites to antigens, supporting the conclusion that CD8^+^ T cells play a critical, direct role in the anti-TB process (Foreman et al., 2016). Our analysis of CDR3 distribution with the D50 index showed clonal expansion exists in CD4^+^ T cells in the TB microenvironment, which is consistent with previous observations (Jasenosky et al., 2015); moreover, we found that CD4^+^ T cell clonal expansion is mainly composed of low- and middle-low frequency CDR3 clonotypes in TPE and blood. Additionally, our sequencing and flow cytometry experimental data further confirmed that the elevated percentages of TRBV4-1^+^CD3^+^ T cells appeared in TPE, suggesting that TRBV4-1 might be of importance in the differential diagnosis of TPE in the clinical setting.

Although a recent study revealed that CD4^+^ CTLs show marked clonal expansion heterogeneously across individuals with dengue virus infection (Patil et al., 2018), thus far, the role of CD4^+^ CTLs during *Mtb* infection has not been fully understood. Early studies on the existence of CD4^+^ CTLs induced by *Mtb* were dismissed by long-term *in vitro* stimulation (Ottenhoff et al., 1988; Mutis et al., 1993). Recent studies showed that CD4^+^ CTLs that produce IFN-γ and chemotactic and cytolytic molecules exhibit antimicrobial functions in *Mtb*-infected individuals (Bastian et al., 2008; Aerts et al., 2019). With the help of single-cell sequencing to describe T cell immunity in human TPE, we confirmed the existence of a subset of polycytotoxic CD4^+^ CTLs, in line with previous findings. We also found these cells expanded and preferentially expressed genes related to glycolysis and tissue residence, indicating that the expanded CD4^+^ CTLs showed transcriptional changes in line with further tissue adaptation.

Conventional MHC-restricted T cells responded specifically to foreign peptides and displaying tolerance to self-antigens. Meanwhile, CD1-restricted T cells, responded to alterations to lipid-related pathways. Guo et al. (2018) found that naive CD4^+^ T cells was activated after stimulated by CD1c^+^ APC presenting lipids and preferentially expressed TRBV4-1^+^ TCRs, which was confirmed as CD1c-restricted autoreactivity (Felley and Gumperz, 2016). Studies have shown that bacterial phospholipids can activate CD1-restricted T cells by binding directly to CD1 from antigen-presenting cells and TCRs, which preferentially express TRBV4-1^+^ TCRs, thus emphasizing a mechanism for CD1-mediated T cell activation to protect against *Mtb* infection (Kasmar et al., 2011; Van Rhijn and Moody, 2015; Shahine et al., 2017; Guo et al., 2018; Reinink et al., 2019; Tezera et al., 2020). A major obstacle in clarifying the *in vivo* relevance of CD1-restricted T cells is the lack of group 1 CD1 molecules in mice, the prevalent animal model for studying immune responses in TB. Lopez et al. (2020) observed that no matter in subjects with Mtb exposure, latent Mtb infection or active TB disease, there were no significant differences in rates of CD1b tetramer, while the frequencies of CD1b tetramers in lower mycobacterial antigen exposure control population were significantly lower. These data indicated that once exposed to mycobacteria, the number of CD1b-specific T cells would increase. However, the authors did not conduct an in-depth study on the characteristics of these cells that are elevated in number after exposure to bound antigen (Lopez et al., 2020). By stimulating human blood cells *in vitro*, Busch et al. (2016) found that the CD1-restricted CD8^+^ T CTL subset expressing IFN-γ and co-expressing three cytotoxic molecules (including granulysin, granzyme B, and perforin) could limit the intracellular growth of *Mtb*, therefore contributing to protection against *Mtb*. Indeed, we found that CD1-restricted CD4^+^ and CD1-restricted CD8^+^ T cells showed T_H_1 and cytotoxic characteristics in TPE patients. However, CD1-restricted CD4^+^ CTLs had not been identified until we characterized this subset in the present study. CD1-restricted CD4^+^ CTLs fulfill several criteria required for an efficient immune response against *Mtb*: (1) activate CD1-mediated T cells by phospholipids derived from *Mtb*; (2) release macrophage-activating T_H_1 cytokines and chemokines required for local immune responses; and (3) produce cytotoxic cytokines to kill *Mtb*. Our results suggest that the induction of the glycolipid–specific CD4^+^ CTL response might represent a novel mechanism of protective immunity against *Mtb*.

This study provides insights into the characteristics and functions underlying T cell response in *Mtb* infectious sites, however, it has several limitations. Besides, although we noticed that both highly enriched CD4^+^ or CD8^+^ T cells expressed higher level of TRBV4-1 than that in non-enriched CD4^+^ or CD8^+^ T cells in TPE by using sc-RNA seq, we found there were no significant differences in the frequencies of TRBV4-1^+^ CD4^+^ or CD8^+^ T cells in enriched T cells with paired TCRαβ sequences between TPE and blood by sc-TCR data. Lacking detection of certain VDJ haplotypes of TCRβ on T cells at the protein level, we also do not know whether CD4^+^, or CD8^+^ T cells with certain VDJ haplotypes, whose TRBV region is TRBV4-1, are the main source of T cell expansion in TB sites.

Still, we have described an unbiased and comprehensive analysis of T cell repertoires in patients with exudative PE that are illustrative of the entire spectrum of T cell adaptive immune response to *Mtb* in the pleural space. We have also identified the existences of polyfunctional CD4^+^ T cells with CD1-restricted, T_H_1 and cytotoxic characteristics in TPE patients, that suggest future vaccine research and development may explore the use of lipid antigens to generate polycytotoxic T cells for enhanced protection. These data further provide insights into the mechanisms of T cell response in exudative PE progression that may have implications for therapeutic interventions.

## Data Availability Statement

The raw sequence data reported in this study have been deposited in the Genome Sequence Archive of the Beijing Institute of Genomics, Chinese Academy of Sciences, under accession numbers HRA000098 and HRA000153 (https://bigd.big.ac.cn/gsa). Other supporting raw data are available from the corresponding author upon reasonable request.

## Ethics Statement

The studies involving human participants were reviewed and approved by the Institutional Review Boards of Beijing Chao-Yang Hospital, Capital Medical University (No. 2018-ke-327), the Wuhan Pulmonary Hospital, Wuhan Institute for Tuberculosis Control (No. 2019-1), and the Nanning Fourth People’s Hospital [No. (2019)28]. The patients/participants provided their written informed consent to participate in this study.

## Author Contributions

M-MS and F-SY designed and analyzed the data and drafted the manuscript. Z-YH assisted to perform the single-cell sequencing. PP and F-YW recruited the subjects. KZ and H-ZS conceived the idea, supervised the study, designed the experiments, analyzed the data, critically revised the manuscript, and guarantee the study’s integrity. All authors read, critically revised, and approved the final manuscript.

## Conflict of Interest

The authors declare that the research was conducted in the absence of any commercial or financial relationships that could be construed as a potential conflict of interest.

## Publisher’s Note

All claims expressed in this article are solely those of the authors and do not necessarily represent those of their affiliated organizations, or those of the publisher, the editors and the reviewers. Any product that may be evaluated in this article, or claim that may be made by its manufacturer, is not guaranteed or endorsed by the publisher.
