# Supplementary material for: T Cell Receptor Repertoire Analysis Reveals Signatures of T Cell Responses to Human Mycobacterium tuberculosis
Source: Front Microbiol. 2022 Feb 7;13:829694. doi: 10.3389/fmicb.2022.829694 (PMC8859175; doi:10.3389/fmicb.2022.829694)
Supplement: Supplementary file 1 [file Data_Sheet_1.PDF]

Supplementary materials

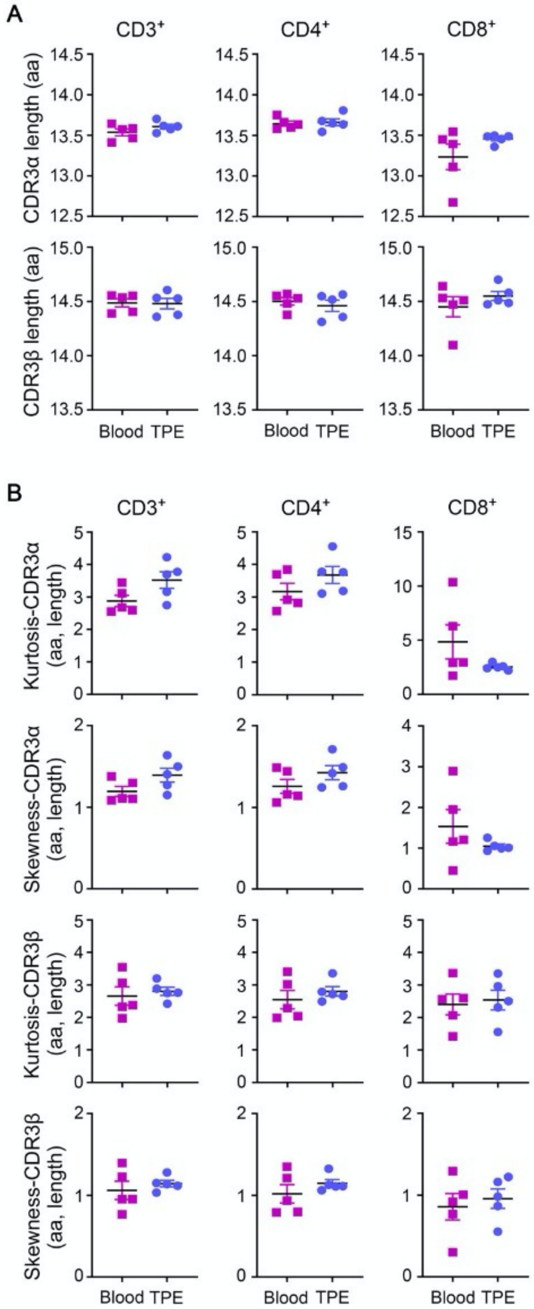

Fig. S1. Comparisons of the CDR3 sequences in $CD3^+$, $CD4^+$, and $CD8^+$ T cells between human TPE and blood. Average length (A), Kurtosis and Skewness index (B) of length distribution of CDR3α and CDR3β aa sequences. Data are presented as means ± SEM. All $P > 0.05$, determined by paired t test.

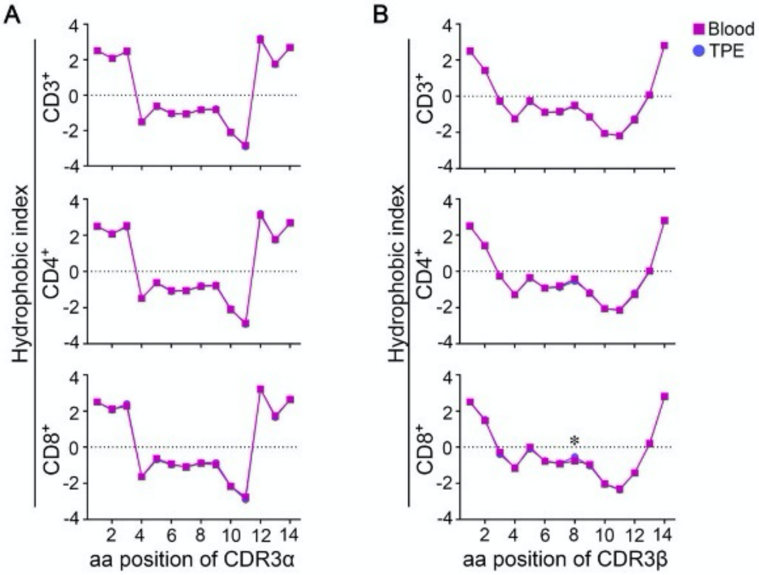

Fig. S2. Comparison of CDR3α and CDR3β region hydrophobicity between human TPE and blood. Hydrophobicity index of CDR3α and CDR3β region in $CD3^+$ (A), $CD4^+$ (B), and $CD8^+$ T cells (C). Position signifies successive aa location at the CDR3 region. *$P < 0.05$, determined by paired t test.

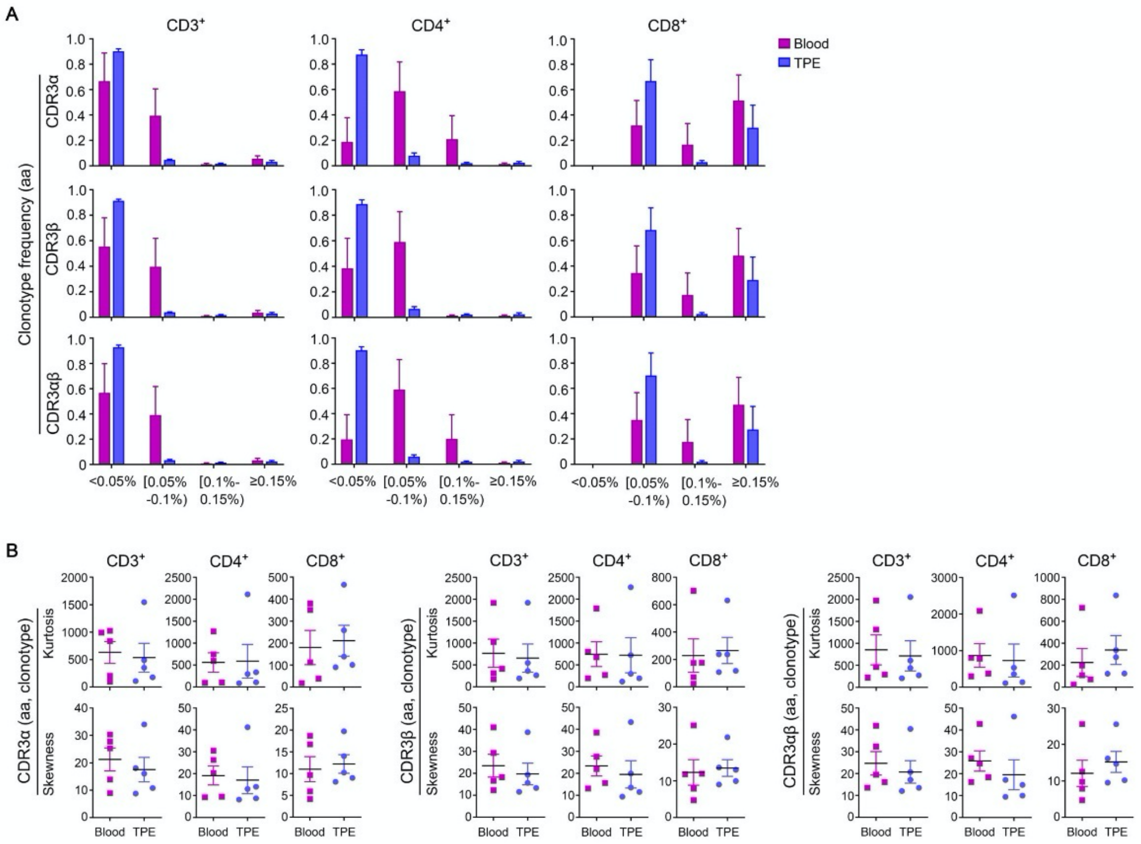

Fig. S3. Comparisons of the CDR3α, CDR3β, and CDR3αβ clonotype frequencies in $CD3^+$, $CD4^+$, and $CD8^+$ T cells between human TPE and blood. Distribution (A), Kurtosis and Skewness index (B) of CDR3 aa clonotype frequencies. Data are presented as means ± SEM. All $P > 0.05$, determined by paired t test.

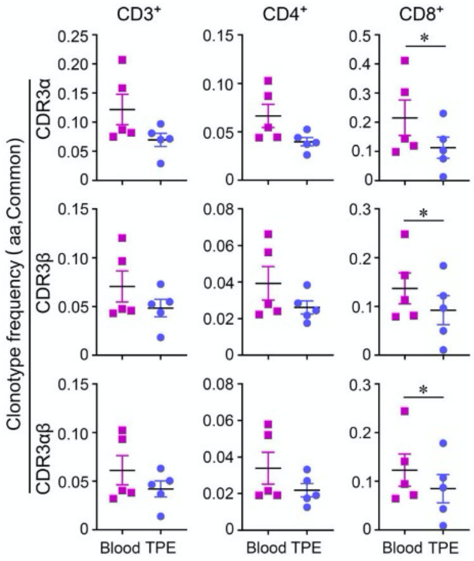

Fig. S4. Comparisons of common TCR aa clonotype frequencies of CDR3α, CDR3β, and CDR3αβ in $CD3^+$, $CD4^+$, and $CD8^+$ T cells between human TPE and blood. Data are presented as means ± SEM. *$P < 0.05$, determined by paired t test.

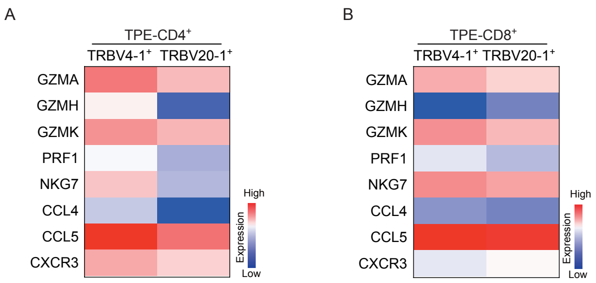

Fig. S5. Cytotoxic genes in TRBV4-1$^+$ T cells and TRBV20-1$^+$ T cells. Expression of cytotoxic genes from TRBV4-1$^+$CD4$^+$ T cells, TRBV20-1$^+$CD4$^+$ T cells (A), TRBV4-1$^+$CD8$^+$ T cells, and TRBV20-1$^+$CD8$^+$ T cells (B) subsets by scTCR-seq and sc-RNA seq.

Table S1. Metric information for single-cell TCR sequencing in 20 samples

Patient	P1		P2		P3		P4		P5	
Sample type	Blood	TPE	Blood	TPE	Blood	TPE	Blood	TPE	Blood	TPE
Read pairs	33,782,353	36,314,809	36,378,521	37,582,966	37,561,579	36,000,039	40,813,649	42,209,837	35,953,418	35,133,770
Cells	2,943	9,131	2,183	6,264	4,788	7,874	5,718	6,142	4,815	8,973
Mean read pairs per cell	11,478	3,977	16,664	5,999	7,844	4,572	7,137	6,872	7,466	3,915
Number of cells with productive V-J spanning pair	2,114	6,943	1,517	4,969	3,536	6,075	4,366	5,175	3,519	7,274
Reads mapped to any V(D)J gene, %	70.1	76.6	81.1	85.3	90.2	91.0	84.3	86.3	84.5	84.9
Reads mapped to TRA, %	17.6	21.4	22.1	26.3	23.9	25.8	16.2	17.7	27.8	29.3
Reads mapped to TRB, %	37.7	42.0	48.1	49.3	58.4	56.7	57.9	58.5	46.9	45.3
Median TRA UMIs per cell	2	2	2	3	3	3	3	4	3	3
Median TRB UMIs per cell	5	6	6	9	9	9	10	10	7	7
Cells with productive V-J spanning pair, %	71.8	76.0	69.5	79.3	73.9	77.2	76.4	84.3	73.1	81.1
Cells with productive V-J.	71.8	76.0	69.5	79.3	73.9	77.2	76.4	84.3	73.1	81.1

spanning (TRA, TRB) pair, %										
Paired clonotype diversity	1724.15	6256.36	195.5	1566.78	1475.14	3516.5	683.08	3127.48	669.33	5028.61
Cells with TRA contig, %	82.5	87.9	82.1	91.2	84.2	89.5	85.3	92.9	85.2	92.8
Cells with TRB contig. %	99.4	99.0	99.3	99.5	99.5	99.3	99.7	99.6	99.2	99.3
Cells with CDR3-annotated TRA contig, %	76.0	80.6	74.7	83.7	77.8	81.1	79.2	87.7	77.8	86.4
Cells with CDR3-annotated TRB contig, %	92.8	94.7	85.9	94.2	86.0	94.2	89.0	95.8	83.3	94.6
Cells with V-J spanning TRA contig, %	79.3	84.1	78.0	87.1	80.8	84.9	81.8	90.1	80.5	89.9
Cells with V-J spanning TRB contig, %	93.7	95.8	88.0	95.7	87.4	95.3	90.3	97.0	85.6	96.5
Cells with productive TRA contig, %	75.1	79.4	73.2	82.4	76.9	79.8	78.5	86.7	76.9	85.0
Cells with productive TRB contig, %	92.4	94.2	85.6	93.9	85.4	93.8	88.8	95.6	82.9	94.2
Patient	P6		P7		P8		P9		P10	

Sample type	Blood	non-TB effusion	Blood	non-TB effusion	Blood	non-TB effusion	Blood	non-TB effusion	Blood	non-TB effusion
Read pairs	43,808,682	45,258,635	52,778,337	37,666,466	37,338,322	38,442,099	33,277,337	34,974,115	55,918,909	52,016,897
Cells	2,878	2,985	2,811	4,522	4,545	5,462	5,686	7,177	2,613	5,866
Mean read pairs per cell	15,221	15,162	18,775	8,329	8,215	7,038	5,852	4,873	21,400	8,867
Number of cells with productive V-J spanning pair	1,337	2,392	2,382	3,924	2,637	4,438	4,159	6,228	2,273	4,945
Reads mapped to any V(D)J gene, %	87.9	92.1	73.7	76.0	80.8	87.2	79.8	82.1	58.8	67.4
Reads mapped to TRA, %	21.8	23.7	19.7	20.1	20.8	22.3	22.0	25.6	17.5	20.2
Reads mapped to TRB, %	58.4	61.7	36.6	35.4	48.7	56.5	47.4	46.1	40.9	47.0
Median TRA UMIs per cell	1	3	5	5	2	3	3	5	5	4
Median TRB UMIs per cell	6	9	9	11	7	9	8	11	12	11
Cells with productive V-J spanning pair, %	46.5	80.1	84.7	86.8	58.0	81.3	73.1	86.8	87.0	84.3
Cells with productive V-J spanning (TRA, TRB) pair, %	46.5	80.1	84.7	86.8	58.0	81.3	73.1	86.8	87.0	84.3

Paired clonotype diversity	87.08	961.3	13.84	5.51	286.32	3691.13	24.63	667.22	598.6	2485.82
Cells with TRA contig, %	54.8	90.0	94.2	96.8	67.6	90.2	83.8	94.3	91.4	90.4
Cells with TRB contig, %	99.6	99.5	99.4	99.5	99.4	99.6	99.4	99.4	99.2	99.1
Cells with CDR3-annotated TRA contig, %	49.9	84.4	88.8	90.0	61.0	84.3	75.9	89.6	89.7	87.7
Cells with CDR3-annotated TRB contig, %	54.6	95.3	92.7	96.1	66.8	97.3	86.0	96.9	98.8	98.7
Cells with V-J spanning TRA contig, %	51.1	87.5	90.7	92.8	62.5	87.8	78.4	91.8	90.7	89.4
Cells with V-J spanning TRB contig, %	55.8	96.8	93.9	96.9	67.7	98.2	87.5	97.9	98.9	98.8
Cells with productive TRA contig, %	49.0	83.0	87.9	88.9	60.2	83.1	75.5	88.8	88.4	86.0
Cells with productive TRB contig, %	54.5	95.2	92.4	95.8	66.5	97.1	85.8	96.7	98.6	98.3

TPE: tuberculous pleural effusion; non-TB: non-tuberculous.

Table S2. Metric information for T cells by merging single-cell TCR sequencing and single-cell RNA sequencing data in 20 samples

Patient	P1		P2		P3		P4		P5		Total
Sample Type	Blood	TPE	Blood	TPE	Blood	TPE	Blood	TPE	Blood	TPE	
Cell number											
$CD3^+$ T cells	1,815	6,504	1,181	4,640	3,033	5,631	3,788	4,985	3,002	7,139	41,718
$CD4^+$ T cells	1,581	4,621	943	4,045	1,941	3,725	3,003	3,727	1,920	5,009	30,515
$CD8^+$ T cells	234	1,883	238	595	1,092	1,906	785	1,258	1,082	2,130	11,203
Patient	P6		P7		P8		P9		P10		Total
Sample Type	Blood	non-TB effusion	Blood	non-TB effusion	Blood	non-TB effusion	Blood	non-TB effusion	Blood	non-TB effusion	
Cell number											
$CD3^+$ T cells	590	2,086	1,837	3,764	1,997	4,108	1,576	5,240	1,692	4,542	27,432
$CD4^+$ T cells	524	1,839	892	1,059	1,888	3,845	1,273	4,258	1,537	3,926	21,041
$CD8^+$ T cells	66	247	945	2,705	109	263	303	982	155	616	6,391

TPE: tuberculous pleural effusion; non-TB: non-tuberculous.

Table S3. Characteristics of lymphocyte immunophenotyping among 10 pleural effusion patients in single-cell sequencing experiment*

	TPE patients (male/female: 3/2)		non-TB effusion patients (male/female: 4/1)	
	Blood	TPE	Blood	non-TB effusion
WBC counts, $\times 10^9$/L	6.3 ± 0.7	4.0 ± 0.9	6.8 ± 1.1	2.9 ± 0.6
Lymphocytes (%)	18.2 ± 1.9	83.6 ± 3.0†	23.2 ± 4.3	80.4 ± 6.6‡
CD4 (% T cells)*	78.4 ± 4.6	73.9 ± 3.6	80.7 ± 8.3	75.5 ± 12.0
CD8 (% T cells)*	25.2 ± 4.6	26.1 ± 3.6	19.3 ± 8.3	24.5 ± 12.0
CD4/CD8*	3.6 ± 0.9	3.3 ± 0.9	8.1 ± 2.8	6.6 ± 2.3

*Annotated by merging single-cell TCR sequencing and single-cell RNA sequencing data. †$P < 0.001$, compared with blood of TPE patients by paired t test. ‡$P < 0.001$, compared with blood of non-TB effusion patients by paired t test.

TPE: tuberculous pleural effusion; non-TB: non-tuberculous
